# Vac8 Controls Vacuolar Membrane Dynamics during Different Autophagy Pathways in *Saccharomyces cerevisiae*

**DOI:** 10.3390/cells8070661

**Published:** 2019-06-30

**Authors:** Fahd Boutouja, Christian M. Stiehm, Christina Reidick, Thomas Mastalski, Rebecca Brinkmeier, Fouzi El Magraoui, Harald W. Platta

**Affiliations:** 1 Biochemie Intrazellulärer Transportprozesse, Ruhr-Universität Bochum, 44780 Bochum, Germany; 2 Biomedizinische Forschung, Leibniz-Institute for Analytical Sciences (ISAS e.V.), 44139 Dortmund, Germany

**Keywords:** autophagy, pexophagy, ribophagy, bulk autophagy, vacuole, Vac8

## Abstract

The yeast vacuole is a vital organelle, which is required for the degradation of aberrant intracellular or extracellular substrates and the recycling of the resulting nutrients as newly available building blocks for the cellular metabolism. Like the plant vacuole or the mammalian lysosome, the yeast vacuole is the destination of biosynthetic trafficking pathways that transport the vacuolar enzymes required for its functions. Moreover, substrates destined for degradation, like extracellular endocytosed cargoes that are transported by endosomes/multivesicular bodies as well as intracellular substrates that are transported via different forms of autophagosomes, have the vacuole as destination. We found that non-selective bulk autophagy of cytosolic proteins as well as the selective autophagic degradation of peroxisomes (pexophagy) and ribosomes (ribophagy) was dependent on the armadillo repeat protein Vac8 in *Saccharomyces cerevisiae*. Moreover, we showed that pexophagy and ribophagy depended on the palmitoylation of Vac8. In contrast, we described that Vac8 was not involved in the acidification of the vacuole nor in the targeting and maturation of certain biosynthetic cargoes, like the aspartyl-protease Pep4 (PrA) and the carboxy-peptidase Y (CPY), indicating a role of Vac8 in the uptake of selected cargoes. In addition, we found that the hallmark phenotype of the *vac8*Δ strain, namely the characteristic appearance of fragmented and clustered vacuoles, depended on the growth conditions. This fusion defect observed in standard glucose medium can be complemented by the replacement with oleic acid or glycerol medium. This complementation of vacuolar morphology also partially restores the degradation of peroxisomes. In summary, we found that Vac8 controlled vacuolar morphology and activity in a context- and cargo-dependent manner.

## 1. Introduction

Yeast cells contain vacuoles that are functionally comparable to mammalian lysosomes and plant vacuoles [1,2]. The defined protein composition of the vacuolar membrane and lumen enables its defined function in protein degradation, metabolite storage, detoxification, and nutrient recycling. The analysis of yeast vacuolar protein sorting mutants has provided general insights into vacuolar membrane traffic and vacuolar biogenesis, which has been a model for organelle inheritance as well as the biosynthetic, endocytic, and autophagic pathways in higher eukaryotes.

One of the central factors is Vac8, which is linked to several functions, even though its molecular mode of action is not known. Vac8 is an actin-associated armadillo repeat protein, which is closely related to plakoglobin and beta-catenin of higher Eukaryotes [3,4,5]. Several different tasks were assigned to Vac8 in *Saccharomyces cerevisiae*. It is required for caffeine resistance, the formation of nucleus–vacuole junctions, homotypic vacuole fusion, cytosol-to-vacuole transport (CVT pathway), and the inheritance of vacuoles [4,5,6,7,8,9]. Some of these functions are defined by the interaction of Vac8 to specific proteins. Vac8 and the phospho-protein Vac17 form a receptor complex for the type V motor protein Myo2 at the vacuole, which is required for the movement of vacuoles to the daughter cell [10]. The interaction of Vac8 with the nuclear membrane protein Nvj1 establishes nucleus–vacuole junctions [8,11]. In the context of the CVT pathway, Vac8 interacts with the phospho-protein Atg13 [6]. Vac8 exhibits one myristoylation and three palmitoylation sites, which contribute to the anchoring of Vac8 at the membrane. It is known that this posttranslational modification is required for vacuolar inheritance and fusion, but not for the CVT pathway [5,6,7,12,13,14,15,16]. In this study, we describe additional functional roles of Vac8 in different autophagic pathways and membrane dynamics in *S. cerevisiae*.

## 2. Materials and Methods

### 2.1. Yeast Strains and Culture Conditions

The *S. cerevisiae* BY4742 strain [17] and its deletion mutants used in this study were purchased from EUROSCARF (Frankfurt, Germany). Yeast complete (YPD; 1% yeast extract, 2% peptone, and 2% glucose; pH 7.4), selective minimal glucose media (SD; 0.3% glucose, 0.5% ammonium sulfate, and 0.17% yeast nitrogen base without amino acids, auxotrophic amino acids, and nucleoside; pH 6.0), and synthetic glucose media (SD(+N); 2% glucose, 0.5% ammonium sulfate, and 0.17% yeast nitrogen base without amino acids, auxotrophic amino acids, and nucleoside; pH 6.0) have been used. Glycerol-containing medium (2% glycerol, 0.1% glucose, 0.5% ammonium sulfate, and 0.17% yeast nitrogen base without amino acids, auxotrophic amino acids, and nucleoside; pH 6.0) and oleate-containing medium (0.5% ammonium sulfate, 0.17% yeast nitrogen base without amino acids, auxotrophic amino acids, and nucleoside, 0.05% Tween40, and 0.1% oleic acid; pH 6.0) have been described previously [18]. In addition to oleate media, the oleate plates contained 0.1% yeast extract, 0.5% Tween40, and 2.4% agar. The nitrogen-starvation medium SD(−N) contained 2% glucose and 0.17% yeast nitrogen base without amino acids, auxotrophic amino acids, and nucleoside, adjusted to pH 6.0.

### 2.2. Plasmids

The Pex11-GFP was generated by amplification of 794 nucleotides upstream of PEX11 and the PEX11 open reading frame without the stop codon by PCR, using genomic *S. cerevisiae* (BY4742) DNA as a template and RE3850 (5′-AAAGAGCTCAAGAAGCTCAAATGAGCGGTT-3′) and RE3985 (5′-AAAGGATCCTGTAGCTTTCCACATGTCTTG-3′) as primers. The PCR product was digested with *Sac*I/*Bam*HI and cloned into pUG35, replacing the MET25 promotor.

The plasmid Pgk1-GFP was a kind gift by Prof. Michael Thumm (Universität Göttingen, GER) [19]. The Pep4-GFP plasmid was provided by Prof. David Goldfarb (University of Rochester, NY, USA) [20]. Rpl25-GFP was ordered from Prof. Michael Rout (Rockefeller University, New York City, NY, USA) via Addgene (Addgene plasmid #24037). The integrating Vac8 and Vac8(C4/5/7A) plasmids were obtained from Prof. Christian Ungermann (Universität Osnabrück, GER) [15].

### 2.3. Bulk Autophagy Assay

To monitor bulk autophagy based on [21], yeast cells expressing the cytosolic protein Pgk1 (3-phosphoglycerate kinase 1) C-terminally fused with GFP were grown in three precultures (first preculture in 10 mL, second and third in 20 mL, OD_600nm_ = 0.1) under selective minimal glucose conditions at 30 °C. In order to initiate bulk autophagy, the third preculture (incubated for 16 h at 30 °C) were washed two times and resuspended in 1 mL sterile dH_2_O. In total, 200 µL cell suspension was transferred to 20 mL SD-medium, which was treated with a final concentration of 0.2 µg/mL rapamycin (Sigma-Aldrich) and/or DMSO as a control. As a starting point of bulk autophagy (t = 0 h), the remaining of cell suspension were immediately harvested at 4000 rpm for 5 min at 4 °C and prepared by TCA precipitation. After 23 h incubation at 30 °C, the t = 23 h samples were harvested (5 min, 4000 rpm at 4 °C), washed two times, and then subjected to TCA precipitation (as described in [22]).

### 2.4. Pexophagy Assay

For the pexophagy assay based on [21], yeast strains expressing the peroxisomal membrane protein Pex11 C-terminally fused with GFP were grown in two precultures (20 mL overnight and 50 mL for 8 h, OD_600nm_ = 0.3) in SD-medium at 30 °C. Peroxisomal proliferation was induced by incubating the cells for 16 h at 30 °C (OD_600nm_ = 0.5) in 100 mL oleate media. To induce pexophagy, the cells first had to be harvested at 4000 rpm for 5 min at 4 °C and washed two times with 5 mL sterile dH_2_O (5 min, 4000 rpm, 4 °C). Cells were resuspended in 1 mL sterile water, and 0.5 mL of cell suspension was transferred to 100 mL nitrogen-starvation media SD(−N). Samples of the starting point (t = 0 h) were taken immediately, harvested for 5 min at 4000 rpm and prepared by TCA precipitation. The culture was incubated for 23 h at 30 °C. After 23 h, the t = 23 h samples (50 mL) were harvested, washed two times and finally precipitated by TCA (trichloroacetic acid) (as described in [22]).

### 2.5. Ribophagy Assay

The ribophagy assay was performed as previously described in [21], with slight modifications. Yeast cells expressing Rpl25-GFP were incubated in 10 mL SD medium for 8 h at 30 °C. The cells (OD_600nm_ = 0.1) were transferred to 20 mL SD(+N) medium, in which they were incubated for 16 h. Then, the cells (OD_600nm_ = 0.3) were transferred to 20 mL SD(+N) medium and were incubated for 8 h, followed by another culture (OD_600nm_ = 0.1) that was incubated for 12 h. After centrifugation, the cells were washed twice in water and resuspended in 1 mL sterile water. To obtain the t = 0 h samples, 0.5 mL of cell suspension were TCA precipitated. In order to induce the degradation of ribosomes, 0.5 mL cell suspension were transferred in 20 mL SD(−N) medium and incubated for 6 h at 30 °C. Finally, the t = 6 h samples were also TCA precipitated (as described in [22]).

### 2.6. Immunodetection

Polyclonal rabbit antibodies were raised against Por1 [23], Pep4 (a kind gift of Prof. Wolf, Stuttgart) and Cpy1 (Abcam, Cambridge, UK). Monoclonal mouse antibodies were raised against GFP (Roche, Mannheim). After elimination of unbound primary antibody, the blots were incubated with goat anti-mouse IRDye^®^ 800CW or IRDye^®^ 680RD goat anti-rabbit as secondary antibodies and visualized with the Odyssey^®^ infrared imaging system (LI-COR Bioscience, Bad Homburg) [24].

### 2.7. Fluorescence Microscopy

In total, 1.5 mL culture was pelleted at 4000 rpm for 5 min at room temperature and resuspended in 100 µL of FM4-64/YPD-medium (1/125 volume of 1 mM FM4-64, purchased from Invitrogen Karlsruhe (T3166)). After cultivation for 30 min at 30 °C, the cell culture was washed twice with YPD medium and incubated in 1 mL fresh YPD-medium for 2 h at 30 °C using a rotator and aluminum foil to shield from the light. Before the cells were subjected to microscopy, they were harvested at 4000 rpm for 5 min at room temperature, washed twice with 1× PBS buffer and then resuspended in 40 to 60 µL 1× PBS buffer. The analysis of live cells was performed with a Zeiss Axioplan microscope and deconvolved with AxioVision 4.1 software (Zeiss, Jena).

### 2.8. Statistical Analysis

The intensity of free GFP signals on the Western Blots was calculated by Image Studio Lite, LI-COR Bioscience (*n* = 5). The results are presented as means ± standard deviation (SD). The analysis of variance was performed by use of t-test procedures. A *p*-value of *p* < 0.001(***) was considered as significant.

## 3. Results

### 3.1. The Biosynthetic Transport via the Carboxy-Peptidase Y (CPY) Pathway Occurs in vac8*Δ* Cells

**Figure 1:**
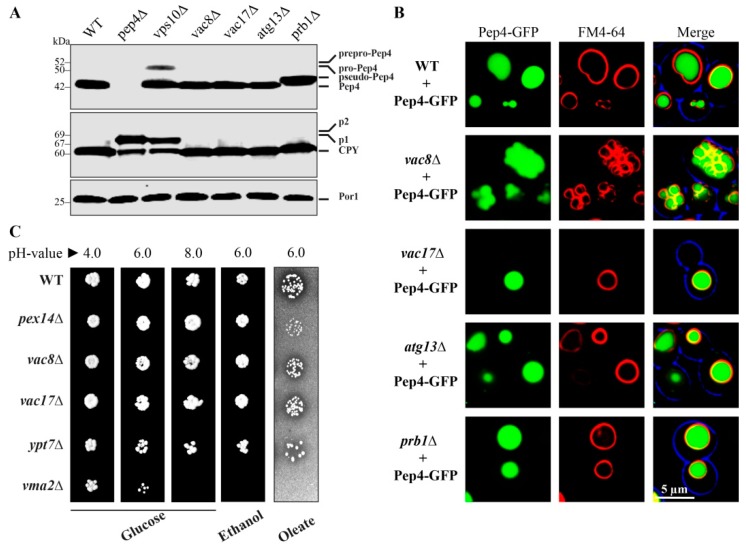
Maturation of the vacuolar proteases Pep4 and Cpy1 is independent of Vac8. (**A**) Fully maturated Pep4 and Cpy1 can be detected in the lysates of wild-type (WT) cells. Non-maturated species can be detected in *vps10*Δ cells, which lack the main trafficking receptor for Pep4 and Cpy1. The strain deleted for the protease Prb1 shows the known pseudo-Pep4, while Cpy1 also displays a species of higher molecular weight. Pep4 and Cpy1 are fully maturated in *vac8*Δ, *vac17*Δ, and *atg13*Δ cells. (**B**) The vacuolar targeting of Pep4-GFP was monitored via the fluorescence microscopy. Pep4-GFP shows a WT-like localization within the lumen of the FM4-64-stained vacuole in *vac8*Δ, *vac17*Δ, *atg13*Δ, and *prb1*Δ cells. (**C**) Vac8 is not involved in the acidification of the vacuole. Vma2 is the subunit B of the V1 peripheral membrane domain of the vacuolar H^+^-ATPase. Therefore, the *vma2*Δ strain does not grow on pH = 8.0 plates. In contrast, vacuolar acidification is not disturbed in WT, *vac8*Δ, *vac17*Δ, and *ypt7*Δ cells. Vac8 is also not essential for the biogenesis of mitochondria and peroxisomes.

It has been described that Vac8 is required for the biosynthetic transport of aminopeptidase 1 (Ape1) to the vacuole via the CVT pathway [6,25]. We were interested to know whether Vac8 also has a role in the Vps10-dependent trafficking pathway of the proteases Pep4 and Cpy1 (CPY pathway). Both Pep4 and Cpy1 are transported as precursor forms that achieve their mature form within the vacuolar lumen [26]. Therefore, we analyzed their maturation status in different mutants (Figure 1A). While wild-type (WT) cells exhibit fully maturated Pep4, the *vps10*Δ cells, which lack the main trafficking receptor, also display a certain amount of precursor Pep4 in addition. The deletion of the protease Prb1 results in the appearance of the known pseudo-Pep4 species [27]. In contrast, we found that *vac8*Δ, *vac17*Δ, and *atg13*Δ cells contained fully maturated Pep4. Neither the Vac8/Vac17 module nor the Vac8/Atg13 module is required for the maturation of Pep4. Similar results were obtained in the analysis of Cpy1. Only *vps10*Δ cells, deleted for the main trafficking receptor, as well as *pep4*Δ cells, deleted for the main activating protease, display an amount of precursor Cpy1. We found that *prb1*Δ cells contain a Cpy1 species that runs at slightly higher molecular weight, indicating that these cells contain a species that could be regarded as a putative pseudo-Cpyl. In contrast, fully maturated Cpy1 was detected in *vac8*Δ, *vac17*Δ, and *atg13*Δ cells, which correlates with our results for Pep4.

We visualized the localization of the Pep4-GFP signal directly with the fluorescence microscope (Figure 1B). The Pep4-GFP signal is properly localized to the lumen of FM4-64 stained vacuoles of WT, *prb1*Δ *vac17*Δ, and *atg13*Δ cells. The fragmented vacuoles of *vac8*Δ cells displayed their known clustered phenotype. However, we found that this did not prevent the import of Pep4-GFP into the lumen of the small, clustered vacuoles.

Because vacuolar enzymes depended on a low pH [28], we tested the acidification of the vacuole in different mutants (Figure 1C). One known critical factor is Vma2 (vacuolar membrane ATPase 2), which is the subunit B of the V1 peripheral membrane domain of the vacuolar H^+^-ATPase, the central electrogenic proton pump [29]. We found that the *vma2*Δ strain grew on glucose medium with pH 4.0 and pH 6.0. However, the shift to the basic pH value of 8.0 is lethal for the *vma2*Δ cells. In contrast, WT and cells lacking Vac8, Vac17, or the vesicle-fusion factor Ypt7 grew on glucose medium with a pH value of 8.0. Therefore, it is strongly suggested that Vac8 does not influence the acidification of the vacuole and that the imported proteases find their optimal pH to be active. Vac8 was also not required for growth on ethanol and oleate at pH 6.0, which indicates that the deletion of VAC8 does not enhance the stress effect of these two media and that it is neither essential for mitochondrial nor peroxisomal biogenesis.

In summary, we found that the vacuolar acidification as well as the Vps10-dependent transport pathway was not affected in *vac8*Δ cells, because they were still capable of the import and maturation of Pep4 and Cpy1.

### 3.2. Vac8 Is Required for Efficient Bulk Autophagy of the Cytosolic Protein Pgk1

**Figure 2:**
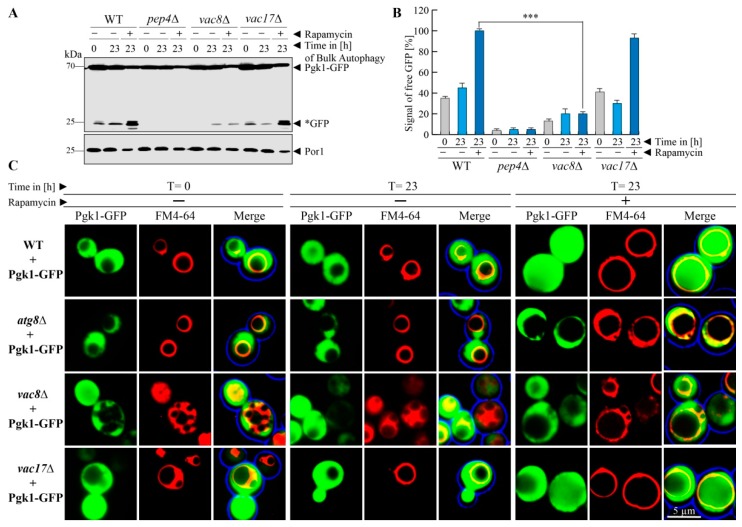
Vac8 is required for efficient bulk autophagy. (**A**) The cytosolic protein Pgk1-GFP shows a certain constitutive turnover. The autophagic degradation of Pgk1-GFP via bulk autophagy was enhanced by the addition of rapamycin. While the Pgk1 portion of the fusion protein is completely degraded in the vacuole, the GFP portion is mostly stable (*GFP). At the end of the bulk autophagy assay (+rapamycin/23 h) a high amount of *GFP was generated in wild-type (WT) and *vac17*Δ cells, while the amount of *GFP did not differ from the −rapamycin/23 h sample in *vac8*Δ cells. (**B**) This result is supported by statistical analysis of the measured densitometry data of the *GFP signals. The amount of *GFP differs significantly between WT and *vac8*Δ cells. *** *p* < 0.001. (**C**) The localization of Pgk1-GFP during bulk autophagy was analyzed by fluorescence microscopy. At the end of the bulk autophagy assay (+rapamycin/23 h), diffuse GFP signals can be detected within the lumen of the FM4-64-stained vacuolar structures of WT and *vac17*Δ cells, while they are devoid of the GFP signal in *atg8*Δ and *vac8*Δ cells.

After the analysis of the trafficking of biosynthetic cargoes, we tested the uptake and breakdown of substrates destined for degradation. We wanted to know whether the degradation of the cytosolic protein Pgk1(3-phosphoglycerate kinase 1)-GFP via bulk autophagy is affected in the *vac8*Δ or *vac17*Δ strain. We used glucose-grown cells and induced bulk autophagy by the addition of the known mTOR-inhibitor rapamycin, which relieves the mTOR-mediated block of autophagic processes [30,31]. This results in the engulfment of cytosolic content by autophagic membranes followed by their degradation in the vacuole. Therefore, the breakdown of the Pgk1-part of the fusion protein should lead to the occurrence of free *GFP, which is relatively stable within the vacuole (Figure 2A). We observe that Pgk1-GFP displays a constitutive turnover, which is significantly enhanced in presence of rapamycin. This was shown by the detection of weak Pep4-dependent *GFP signals at the time point t = 0 h in WT cells, which are clearly enhanced in presence of rapamycin (t = 23 h). Similar results were obtained with *vac17*Δ cells, demonstrating that Vac17 is not involved in bulk autophagy. In contrast, the samples derived from *vac8*Δ cells showed that bulk autophagy was hampered in these cells. The constitutive autophagic turnover of Pgk1-GFP was nearly blocked, as no free *GFP was produced at t = 0 h and only a small portion of free *GFP was detectable at t = 23 h (−rapamycin). This amount of free *GFP did not increase when rapamycin had been added (t = 23 h + rapamycin). The statistical analysis of the densitometric data of the *GFP signal intensities (Figure 2B) in *vac8*Δ cells showed that the amount of *GFP at the end of the bulk autophagy assay (t = 23 h + rapamycin) was even lower than the basic turnover of Pgk1-GFP in WT cells (t = 0 h − rapamycin). In line with this, the amount of free *GFP generated in the bulk autophagy assay (t = 23 h + rapamycin) was significantly lower in *vac8*Δ cells than in WT cells. These results strongly suggest a critical involvement of Vac8 in the autophagic degradation of the cytosolic protein Pgk1-GFP via bulk autophagy.

We further analyzed the requirement of Vac8 for bulk autophagy with the fluorescence microscope (Figure 2C). In WT cells, Pgk1-GFP proteins can be detected as green cytosolic signals, which leave out the vacuole to a certain extent. This corresponds to the situation at t = 0 h during the biochemical assay, when only little Pgk1-GFP is degraded via constitutive turnover.

After the incubation with rapamycin (t = 23 h), the vacuoles were filled with strong green signals, which indicates the uptake and efficient breakdown of the Pgk1-GFP fusion protein in the vacuole. The *atg8*Δ strain served as negative control and contained no vacuolar GFP signals. The *vac17*Δ cells behaved similar to the WT. The analysis of the *vac8*Δ cells revealed that the lumen of the fragmented vacuoles was nearly devoid of GFP signals, which corresponds to the finding from our biochemical assays that Vac8 is required for efficient bulk autophagy of Pgk1-GFP after rapamycin induction.

It is interesting to note that the efficiency of degradation differs depending on the mode of mTOR inhibition. Previous work found that the bulk autophagy cargoes Pho8∆60 and GFP-Atg8 were degraded efficiently when *vac8*Δ cells were grown in starvation medium [6,32]. We also found a compromised but still efficient degradation of Pgk1-GFP in *vac8*Δ cells when we used starvation (SD − N) medium (Figure S1). Therefore, at least for Pgk1-GFP we can say that the presence of Vac8 is obviously more important for Pgk1-GFP breakdown when cells are grown in glucose medium (+rapamycin) than in starvation medium.

### 3.3. Vac8 is Essential for Pexophagy in S. cerevisiae

In order to analyze the selective autophagic degradation of peroxisomes, we utilized the peroxisomal membrane protein Pex11 genetically fused to GFP as marker. As the first step, the proliferation of peroxisomes was induced by the shift of glucose-grown cells to oleate-containing medium, which lacks glucose.

**Figure 3:**
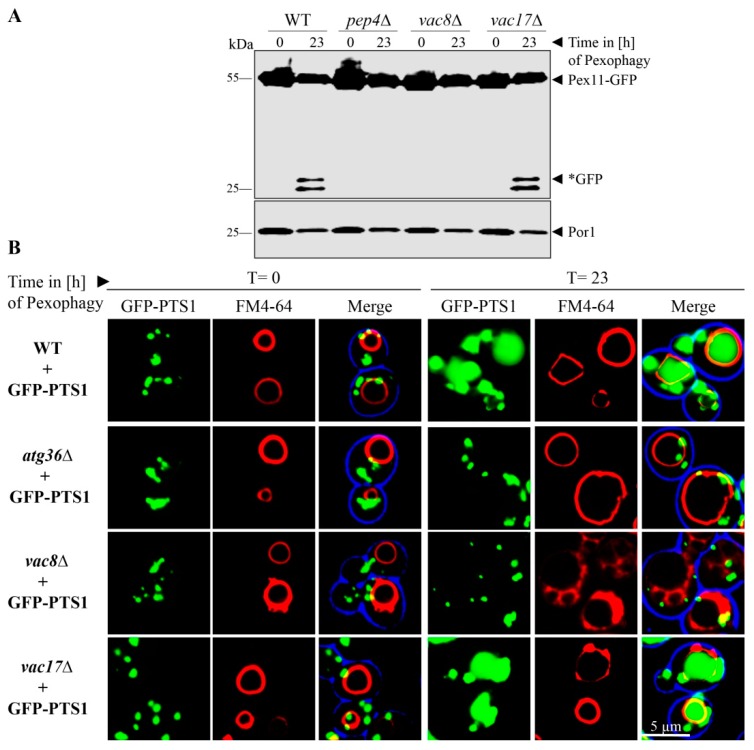
Vac8 is essential for pexophagy. (**A**) The possible contribution of Vac8 and Vac17 to pexophagy was monitored via the stability of the peroxisomal membrane protein Pex11, which was genetically fused to GFP. After proliferation of peroxisomes, cells were shifted to pexophagy medium. In wild-type (WT) and *vac17*Δ cells, the Pex11-part of the fusion protein is degraded in the vacuole together with the rest of the organelle, while the GFP portion is largely stable (*GFP). This process is blocked in *pep4*Δ and *vac8*Δ cells. (**B**) Peroxisomes were labeled with the matrix protein GFP-PTS1 for fluorescence microscopy. The vacuolar membrane was stained with FM4-64. Prior to the induction of pexophagy (t = 0 h), peroxisomes are visible as green cytosolic dots, while the vacuolar lumen is devoid of these signals. At the end of the pexophagy assay (t = 23 h), some peroxisomal signals remain in the cytosol, while the vacuole of WT cells is filled with a diffuse green staining, indicating peroxisomal breakdown. The vacuole does not display GFP signals in *atg36*Δ and *vac8*Δ cells, indicating a defect in pexophagy.

Peroxisomes are essential for growth in oleate medium, and therefore their proliferation is induced under these conditions [18,33,34]. As the second step, the cells were shifted to glucose-containing medium, which lacks oleate and contains a reduced amount of nitrogen sources in order to induce the degradation of the not needed excess of peroxisomes. We found that the peroxisomes marked with Pex11-GFP were degraded in the vacuoles of WT cells (Figure 3A). While Pex11 is hydrolyzed together with the rest of the peroxisome, the GFP-fusion-tag remains relatively stable within the vacuole. Therefore, the occurrence of free *GFP is regarded as a marker for functional pexophagy. In contrast, the deletion of the protease Pep4 served as negative control, because no free *GFP is generated.

The deletion of Vac17 did not inhibit pexophagy. This was shown by the detection of free *GFP in the samples of the cell lysate. In contrast, the deletion of Vac8 resulted in a complete block of peroxisomal breakdown by pexophagy. No free *GFP was detectable in the lysate of *vac8*Δ cells. The mitochondrial Por1 served as loading control and showed that equal amounts were loaded for the t = 0 h test samples as well as for the t = 23 h test samples, respectively.

We verified this result by monitoring pexophagy by fluorescence microscopy (Figure 3B). We transformed the cells with the peroxisomal matrix protein marker GFP-PTS1. The cells were incubated for the pexophagy assay as described above. WT cells display a green punctated pattern at the time point t = 0 h, which corresponds to the peroxisomes with imported GFP-PTS1. At the time point t = 23 h after pexophagy induction, single green dots, representing intact peroxisomes, can still be seen in the cytosol, along with broad, diffuse green signals within FM4-64 stained vacuoles, indicating peroxisomal breakdown by displaying only the remaining free *GFP. The negative control *atg36*Δ, which lacks the peroxisomal pexophagy receptor, displays no GFP signals within the vacuolar lumen. Similar to WT cells, the vacuoles of *vac17*Δ cells contain diffuse green signals. In contrast, the vacuoles of *vac8*Δ cells are devoid of GFP signals after pexophagy induction.

In summary, we could show that Vac8 is essential for the selective autophagic degradation of peroxisomes in oleate-induced cells of *S. cerevisiae*.

### 3.4. Vac8 is Essential for Ribophagy

**Figure 4:**
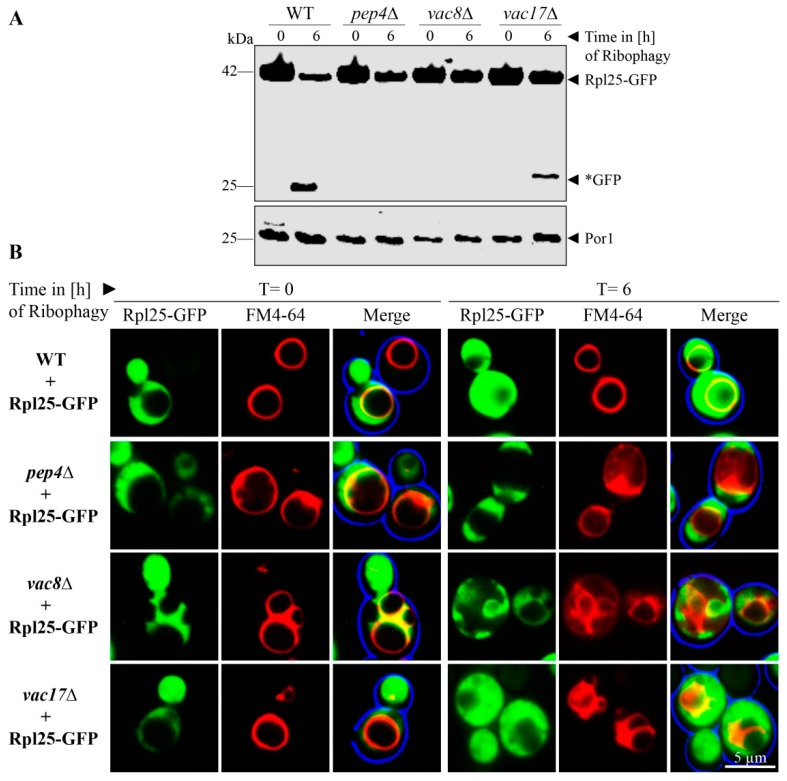
Vac8 is essential for ribophagy. (**A**) The possible contribution of Vac8 and Vac17 to ribophagy was analyzed via the stability of the ribosomal protein Rpl25, which was genetically fused to GFP. In wild-type (WT) and *vac17*Δ cells, the Rpl25-part of the fusion protein is degraded in the vacuole together with the rest of the organelle, while the GFP portion is largely stable (*GFP). This process is blocked in *pep4*Δ and *vac8*Δ cells. (**B**) Ribosomes were labeled with Rpl25-GFP for fluorescence microscopy. The vacuolar membrane was stained with FM4-64. At the end of the ribophagy assay (t = 6 h), the vacuole of WT and *vac17*Δ cells is mainly filled with a diffuse green staining, indicating ribsomal breakdown. The vacuole does not display GFP signals in *pep4*Δ and *vac8*Δ cells, indicating a defect in ribophagy.

Based on our finding that Vac8 is required for the bulk autophagy of the cytosolic protein Pgk1-GFP and essential for the selective autophagic degradation of peroxisomes via pexophagy, we tested the role of Vac8 in the autophagic breakdown of ribosomes. The ribosomal protein of the large subunit Rpl25 (ribosomal protein large 25) was fused to GFP in order to analyze the ribophagy of the 60S subunit (Figure 4A). At the end of the ribophagy assay (t = 6 h), free *GFP was generated in WT and *vac17*Δ cells. In contrast, the *vac8*Δ cells show no *GFP, like the negative control *pep4*Δ.

Ribophagy was also monitored by fluorescence microscopy (Figure 4B). WT cells display green cytosolic signals at the time point t = 0 h, which correspond to the intact ribosomes, while the vacuole is devoid of GFP. At the time point t = 6 h, diffuse green signals can be detected within the FM4-64 stained vacuoles. Similar to WT cells, the vacuoles of *vac17*Δ cells contain diffuse green signals, which correspond to free *GFP derived from degraded ribosomes. The vacuoles of *vac8*Δ cells display no GFP signals within their lumen, strongly suggesting a defect in uptake and degradation of ribosomes during ribophagy.

### 3.5. Palymitoylation of Vac8 Controls Pexophagy and Ribophagy

**Figure 5:**
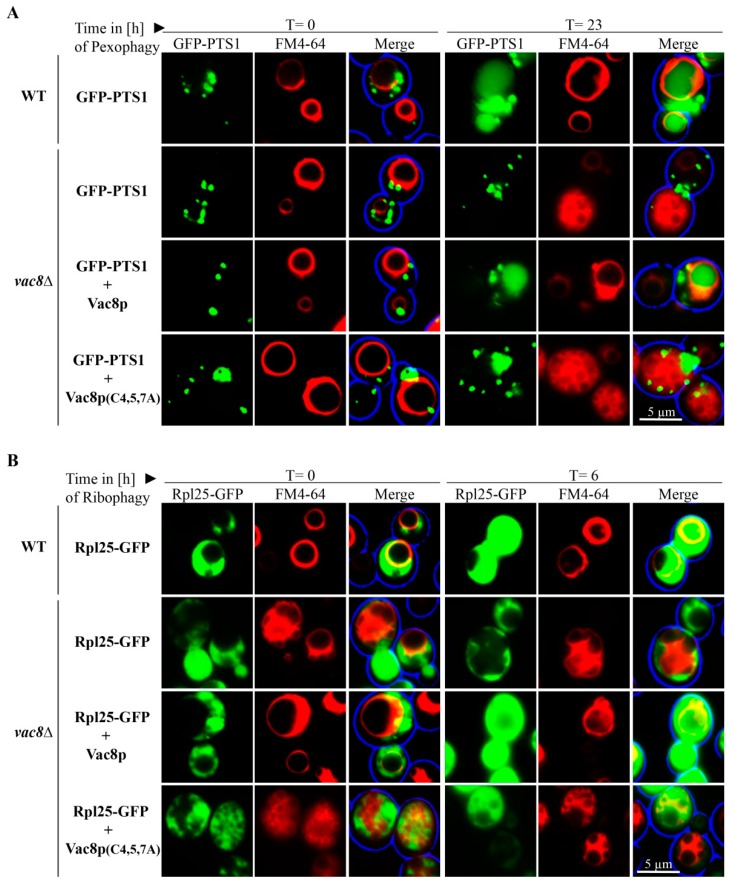
Palmitoylation of Vac8 is required for efficient pexophagy and ribophagy. (**A**) Peroxisomes were labeled with the matrix protein GFP-PTS1, while the vacuolar membrane was stained with FM4-64 for fluorescence microscopy. WT and *vac8*Δ cells transformed with an integrating Vac8 plasmid are filled with a diffuse green staining at the end of the assay (t = 23 h), indicating peroxisomal breakdown. The vacuole does not display GFP signals in *vac8*Δ cells without or with the palmitoylation-deficient Vac8(C4/5/7A), indicating a defect in pexophagy. (**B**) Ribosomes were marked with Rpl25-GFP, while the vacuolar membrane was labeled with FM4-64. At the end of the ribophagy assay (t = 6 h), the vacuoles of WT and *vac8*Δ cells transformed with a Vac8 plasmid were filled with GFP signals, indicating ribosomal degradation. The vacuole does not display GFP signals in *vac8*Δ cells and *vac8*Δ cells transformed with the palmitoylation-deficient Vac8(C4/5/7A), strongly suggesting a defect in ribophagy.

We were interested to analyze whether the palmitoylation of Vac8 has an impact on its role in selective autophagy. We transformed GFP-PTS1 expressing *vac8*Δ cells with integrating plasmids encoding either for wild-type Vac8 or a palmitoylation-deficient version, in which the cysteines at position 4, 5, and 7 have been exchanged against alanines (Figure 5A).

We found that *vac8*Δ cells containing plasmid-encoded Vac8 displayed diffuse vacuolar GFP signals, showing that peroxiosmes were degraded and that the plasmid could complement the deletion strain. In contrast, Vac8(C4/5/7A) containing cells exhibited vacuoles that were nearly completely devoid of GFP signals. This result shows that palmitoylation of Vac8 controls the degradation of peroxisomes via pexophagy.

We also tested the role of the palmitoylation of Vac8 during ribophagy (Figure 5B). Therefore, we transformed Rpl25-GFP expressing *vac8*Δ cells with integrating plasmids encoding either for wild-type Vac8 or Vac8(C4/5/7A). Vacuolar GFP signals can be detected in WT cells and in *vac8*Δ cells containing plasmid-encoded Vac8. In contrast, *vac8*Δ cells without Vac8 plasmids do not exhibit vacuolar GFP, indicating a defect in the uptake and degradation of ribosomes. The *vac8*Δ cells containing the palmitoylation-deficient Vac8(C4/5/7A) displayed vacuoles that appear to be nearly devoid of GFP signals, suggesting that palmitoylation is required for ribophagy. In order to corroborate this finding, we also performed a biochemical ribophagy assay (Figure S2). In line with our fluorescence data, we found that Vac8 containing cells could produce free *GFP, while Vac8(C4/5/7A) containing cells exhibited only hardly detectable amounts of free *GFP.

Another interesting finding concerns the morphology of the vacuole in *vac8*Δ cells. They appear to form WT-like round central vacuoles after oleate induction (Figure 5A, t = 0 h). However, when *vac8*Δ cells or *vac8*Δ cells containing Vac8(C4/5/7A) are grown on glucose-based medium, like the pexophagy medium (Figure 5A, t = 23 h) or with or without rapamycin in ribophagy medium (Figure 5B, t = 0 h/t = 6 h), they appear to be fragmented. Because the size of the vacuolar structures and the degree of fragmentation seem to change under different conditions, we tested the effect of distinct incubation methods systematically.

### 3.6. The Vacuolar Fusion-Defect of Glucose-Grown vac8Δ Cells Can Be Complemented by Oleic Acid and Glycerol

**Figure 6:**
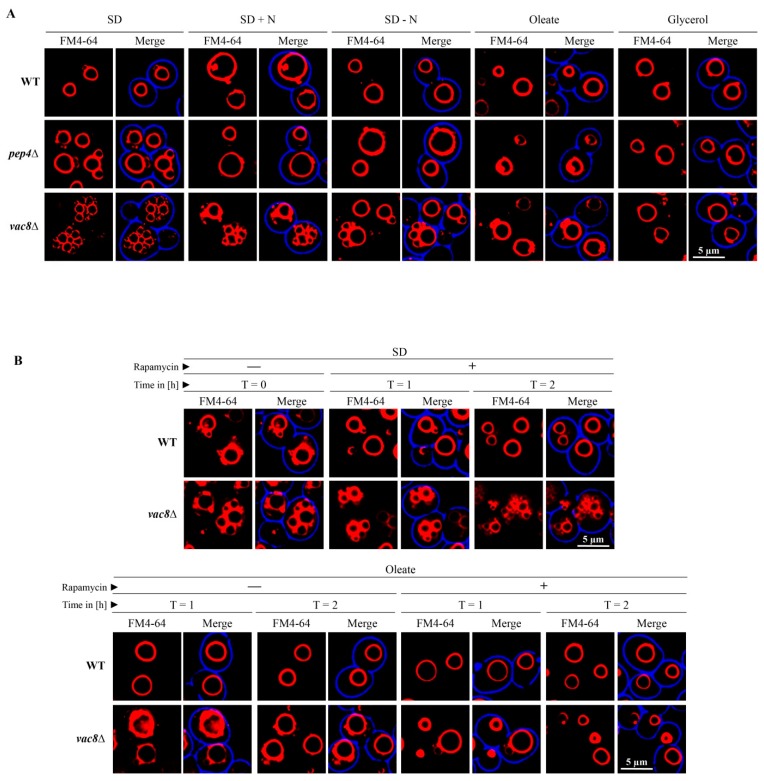
The vacuolar fusion-defect of glucose-grown *vac8*Δ cells can be complemented by oleic acid and glycerol. (**A**) Glucose-grown (SD) *vac8*Δ cells display the typical fragmented vacuolar phenotype, which is thought to be caused by a vacuolar fusion defect. The shift to SD − N medium leads to the formation of a larger central vacuole associated with some smaller vacuolar structures. The shift of SD grown cells to medium containing oleic acid or glycerol resulted in the formation of vacuoles comparable to WT cells. (**B**) The morphology change of *vac8*Δ cells is rapamycin-independent. The morphology of FM4-64-stained vacuoles of cells grown in SD medium is not influenced by the presence of rapamycin under the tested conditions. The presence of rapamycin does not block the morphology shift of *vac8*Δ cells from SD medium to oleic acid medium. The small fragmented vacuoles (t = 0 h) fused to large vacuoles (t = 1 h/t = 2 h) with or without rapamycin.

Glucose-grown (SD medium) *vac8*Δ cells display the typical fragmented vacuolar phenotype (Figure 6A), which is caused by a vacuolar fusion defect [4]. When we shifted the SD-grown cells to a medium with additional nitrogen sources (SD + N) or reduced nitrogen sources (SD − N), the appearance of the vacuolar structures in *vac8*Δ cells changes to certain extent. Nearly no change can be seen in cells growing in SD + N medium, while cells in SD − N medium, a starvation medium, show one slightly more enlarged central vacuole associated with several smaller vacuolar structures.

The shift of SD grown cells to medium predominantly containing oleic acid or glycerol resulted in the formation of one large vacuole that is comparable to WT cells. Therefore, it can be concluded that the vacuolar fusion-defect of glucose-grown *vac8*Δ cells can be complemented by oleic acid and glycerol. The smaller (SD − N) and larger (oleic acid, glycerol) changes in morphology occur quite rapidly and are visible 1 h after medium shift.

We wanted to elucidate whether the morphology change in *vac8*Δ cells is dependent on mTOR activity. First, we tested the effect the mTOR inhibitor rapamycin on cells incubated in SD medium (Figure 6B). We found that the vacuolar morphology did not change in the presence of rapamycin in SD medium under the chosen conditions. Next, we tested whether the oleic acid induced morphology change of *vac8*Δ cells is influenced by mTOR. The results show that the presence of rapamycin does not block the morphology shift of *vac8*Δ cells from SD medium (t = 0 h) to oleic acid medium (t = 1 h/t = 2 h). Therefore, the results suggest that the small fragmented vacuoles visible in SD medium can fuse to one large vacuole in oleic acid medium with or without rapamycin within 1 h.

### 3.7. The Defect of Peroxisome Degradation in vac8Δ Cells Can Be Complemented by Oleic Acid and Glycerol

**Figure 7:**
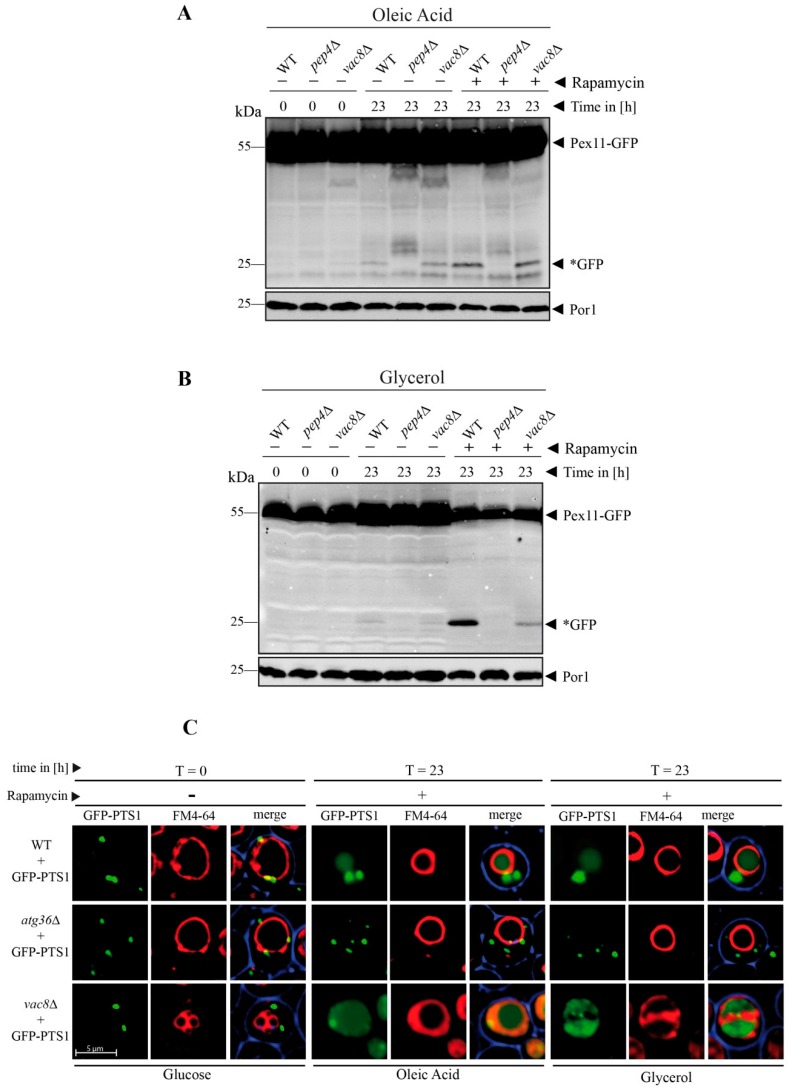
The peroxisome degradation-defect of *vac8*Δ cells can be complemented by oleic acid and glycerol. (**A**) The degradation of peroxisomes is restored in *vac8*Δ cells when the cells are shifted to oleic acid medium. The Pex11-part of the fusion protein is degraded in the vacuole together with the rest of the organelle, while the GFP portion is largely stable (*GFP). This process is blocked in *pep4*Δ. (**B**) The degradation of peroxisomes is partially restored in *vac8*Δ cells when the cells are shifted to glycerol medium. The Pex11 portion is degraded in the vacuole along with the rest of the organelle, while the GFP part is largely stable (*GFP). This process is inhibited in *pep4*Δ. (**C**) Visualization of cellular components under the microscope during the peroxisome degradation assay. Peroxisomes were labeled with GFP-PTS1 and vacuoles were stained with FM4-64. The glucose-grown cells display the fragmented vacuolar phenotype. After the shift to glycerol or oleate, the fusion defect is nearly completely recovered and a large central vacuole can be seen. These vacuoles are able to function in peroxisomal degradation as indicated by the diffuse green staining within the vacuole.

We have found that Vac8 is essential for the degradation of peroxisomes via pexophagy. Because oleic acid and glycerol can bypass the requirement for Vac8 in the fusion process and thereby can complement the fusion defect by enabling the fragmented vacuolar parts to form one central vacuole, we wanted to elucidate whether they could also complement the degradation defect of peroxisomes. We shifted the glucose (SD medium)-grown cells containing Pex11-GFP to oleic acid medium and induced autophagic degradation of peroxisomes via the addition of rapamycin (Figure 7A).

At t = 23 h, the WT cells display free *GFP, indicating the autophagic breakdown of peroxisomes. No *GFP was visible in the *pep4*Δ strain, which served as negative control. In contrast to the previous pexophagy assay (Figure 3), where the *vac8*Δ strain showed no *GFP and therefore was blocked in pexophagy, the *vac8*Δ cells were able to produce a certain amount free *GFP in presence of oleic acid and rapamycin. This result strongly suggests that the addition of oleic acid can not only bypass the requirement of Vac8 for the fusion process (Figure 6) but also at least partially for the peroxisome degradation defect (Figure 7A).

Similarly, the degradation of peroxisomes can be achieved in a *vac8*Δ strain grown in glycerol medium (Figure 7B). Glycerol can not only induce the transition from several fragmented to one large vacuole (Figure 6), but also partially restore the peroxisomal degradation defect of the *vac8*Δ strain (Figure 7B).

A comparable effect was also detected for the degradation of Pgk1-GFP via bulk autophagy (Figure S3). While only very little *GFP was generated in *vac8*Δ cells after rapamycin treatment in glucose medium (Figure 2A), we then found that the *GFP in oleate-grown cells after rapamycin addition reached a WT-like level (Figure S3).

In order to directly coordinate the morphology and activity of the vacuole in *vac8*Δ cells, we performed the pexophagy assay for GFP-PTS1 marked peroxisomes using fluorescence microscopy (Figure 7C). The *vac8*Δ cells show their clustered phenotype when they were grown in glucose medium (t = 0 h). When they were shifted to oleic acid or glycerol medium, they developed larger vacuoles and were able to degrade peroxisomes, as indicated by the diffuse green staining within the vacuole (Figure 7C).

In summary, the peroxisome degradation defect of *vac8*Δ cells can partially be complemented by oleic acid and glycerol, the central two building blocks of membrane lipids.

## 4. Discussion

The armadillo repeat protein Vac8 is closely related to plakoglobin of higher Eukaryotes [3,4,5] and has been assigned to several functions in *S. cerevisiae*. It has been reported to be required for the inheritance of vacuoles, homotypic vacuole fusion and cytosol-to-vacuole transport (CVT) in *S. cerevisiae* [5,6,7]. In our study, we found that Vac8 was also required for the bulk autophagy of cytosolic content and that it is essential for the selective autophagic degradation of peroxisomes via pexophagy and the selective autophagic degradation of ribosomes via ribophagy. The possibility that Vac8 might play an important role in bulk autophagy was not clearly defined until now [5,6,32]. This was based on the finding that the breakdown of the autophagosomal membrane marker GFP-Atg8 was only mildly affected [32] as well as on the finding that the vacuolar proteolysis of the artificial cytosolic cargo Pho8Δ60 (a cytosolic variant of the vacuolar alkaline phosphatase, which lacks the transmembrane domain) was negatively influenced but not blocked by the deletion of *VAC8* [6]. Moreover, electron microscopy data showed vesicles within the vacuoles of *vac8*Δ cells, which were assumed to represent autophagic bodies that were most likely related to bulk autophagy [6], resulting in conclusions that *vac8*Δ cells are not defective in autophagy or that autophagy was still active [5,6]. In contrast, our data directly demonstrate that the deletion of *VAC8* blocks the vacuolar import and efficient degradation of the cytosolic protein Pgk1 fused to GFP in the context of the constitutive turnover as well as after induction of bulk autophagy by rapamycin. We showed this with biochemical as well as fluorescence microscope-based assays. Bulk autophagy is nearly but not completely blocked, because a small amount of free *GFP is detectable with the antibody in cell lysates or as minor green fluorescence signal within the vacuoles of living cells at the end of the assay (23h + rapamycin). Therefore, we can conclude that Vac8 is not essential but clearly required for bulk autophagy.

Pgk1-GFP, GFP-Atg8, and Pho8Δ60 are well-known substrates for bulk autophagy [19,21,35]. One difference is that the reported experiments with the proteolytic cleavage of Pho8Δ60 and GFP-Atg8 as well as the EM data were made after the shift to starvation medium (SD − N), while our experiments with Pgk1-GFP were started with the addition of rapamycin. Even though both conditions should block mTOR activity, the kinetics of the downregulation of the corresponding signaling cascades might differ and result in distinct effects on mTOR downstream factors. Moreover, the different results could potentially be explained by the idea that Pgk1-GFP, GFP-Atg8, and Pho8Δ60 are taken up by different mechanisms, which could reflect different bulk autophagy modes, like micro- and macroautophagy. Further work will have to clarify this question experimentally.

However, what we can say from our experiments is that the medium conditions do have an influence on the degradation efficiency of Pgk1-GFP. Therefore, we also studied the degradation of Pgk1-GFP under starvation SD − N conditions. We found, in basic agreement with most of the Pho8∆60 and GFP-Atg8 data, that Pgk1-GFP can still be degraded relatively efficiently under starvation conditions. Therefore, we can conclude that Vac8 is less important for the degradation of Pgk1-GFP under starvation SD − N conditions, while Vac8 is more required for the efficient degradation of Pgk1-GFP after rapamycin treatment. This is in agreement with our morphological data. We have shown that *VAC8*-deficient cells display the clustered vacuole in glucose medium and that rapamycin does not or only very slowly influence the morphology under the tested conditions. Therefore, Pgk1-GFP is not efficiently degraded in the glucose/rapamycin assay. We have also shown that SD − N medium results in a faster partial reconstitution of the morphology of the large central vacuole. This partial complementation of the fusion defect seems to be sufficient for a relatively efficient degradation of cytosolic proteins like Pgk1-GFP by bulk autophagy in *VAC8*-deficient cells. This is at least the effect for cytosolic proteins, but not sufficient for organelles. The degradation of Pgk1-GFP reaches a WT-like rate when *vac8*Δ vacuoles are the largest under oleic acid conditions.

In addition to our finding that Vac8 is involved in bulk autophagy, we find that it is essential for the selective autophagic degradation of peroxisomes and ribosomes in *S. cerevisiae* in a palmitoylation-dependent manner. Previous work has linked Vac8 specifically to micropexophagy in the methylotrophic yeast *Pichia pastoris* [36,37,38]. It was only required for glucose-induced micropexophagy, but not for macropexophagy. Moreover, Vac8 was only required for the degradation of peroxisomes that had been proliferated by the induction with methanol, but it had no effect on the breakdown of peroxisomes that had been proliferated with ethanol, amines or oleate. In contrast, we find that Vac8 is essential for the degradation of oleate-induced peroxisomes by starvation-induced pexophagy in *S. cerevisiae*. Because so far macro- and micropexophagy cannot be separated in *S. cerevisiae*, the corresponding pexophagy mode is not known. In addition to pexophagy, our finding of a general requirement of Vac8 for ribophagy as well as the involvement in bulk autophagy show that Vac8 has a central role in different autophagy pathways in *S. cerevisiae*. Moreover, we found that pexophagy and ribophagy were regulated by the palmitoylation of Vac8. Another question could concern the role of the myristoylation of Vac8. However, the Vac8(G2A) mutant is expected to behave similar to the Vac8(C4/5/7A) mutant, as suggested by a similar loss of membrane association [5,15].

Our findings concerning the transport of cargoes destined for degradation via different autophagy pathways fit well to the distinct concepts of the biosynthetic transport pathways. It has been reported that the transport of the protease Ape1 via the CVT pathway depends on the presence of Vac8 [5,6]. The CVT pathway shares mechanistic similarities and certain proteinaceous components with the machinery of selective autophagy [39,40]. Therefore, Vac8 might perform a similar function in the CVT pathway as in autophagy. We also tested the import and maturation of Vps10-dependent proteases of the CPY pathway, which is mechanistically not related to autophagy. We found that the transport of the proteases Pep4 and Cpy1 did not require Vac8. These two proteases are not transported via the CVT pathway within spherical CVT complex particles but by the membrane-bound trafficking receptor Vps10 via the carrier vesicles of the early secretory pathway [26]. Therefore, it can be assumed that the function of Vac8 in the regulation of membrane fusion events is selective for certain cargoes, as it is required for the autophagy-related CVT pathway, but not required for the uptake of Vps10-containing transport carriers of the CPY pathway.

The sum of the data indicate that major vacuolar membrane dynamics might be required for the uptake of selective autophagy cargoes (ribophagy, pexophagy) or the cargoes of selective autophagy-like pathways (CVT), while the uptake of non-autophagic vesicular carriers, as we have demonstrated for the Vps10-dependent CPY transport, is independent of Vac8. Thus, although these different autophagy pathways exhibit distinct requirements and partially different signaling factors, they seem to converge at a Vac8-dependent step at the vacuolar membrane or at least share Vac8-dependnet vacuolar membrane dynamics as a prerequisite for the uptake and degradation of their cargoes.

The morphology of the vacuole is responsive to cellular stress. The changes in vacuolar morphology are mediated via an alteration in the equilibrium between fission and fusion processes. The hallmark phenotype of *vac8*Δ cells are their fragmented vacuoles. We show that important building blocks for membrane lipids, namely oleic acid and glycerol, can complement the *vac8*Δ vacuolar fusion defect. The presence of either of these two factors leads to a disappearance of the small fragmented vacuolar structures, resulting in vacuoles with WT-like appearance. As shown in the fluorescence microscopy assays for pexophagy, this process is reversible, as the shift from oleate medium to glucose-based pexophagy media triggers the reappearance of the fragmented phenotype.

Until now, individual membrane lipids have been described to be required for membrane fusion, like diacylglycerol (DAG), phosphatidic acid, ergosterol as well as several phosphoinositol species [41,42]. They are thought to act as fusion protein docking sites or to define membrane microdomains that are involved in the priming for the fusion event by organizing the SNAREs, Ypt7, HOPS, and actin at the site of fusion [41,42].

Concerning single fatty acids, it has been reported that the addition of the activated fatty acid palmitoyl-CoA stimulates the fusion rate of vacuoles in WT cells [43]. Palmitoyl-CoA serves as a source for the palmitoyl moiety of posttranslationally modified proteins. Several proteins involved in vacuolar fusion and membrane trafficking are palmitoylated [44]. In line with this, a link between the synthesis of palmitoyl-CoA and Vac8 has been suggested [16]. The block of fatty acid synthesis in the conditional mutant of Acc1 (acetyl-CoA carboxylase 1) results in a loss of Vac8 palmitoylation and a *vac8*Δ-like phenotype of the vacuolar morphology [16]. Therefore, the general addition of palmic acid seems to trigger fusion via protein modifications like the palymitoylation of Vac8. In contrast, Vac8 is not modified by oleic acid, and therefore our finding of a role of oleic acid in vacuolar fusion requires a different explanation. Oleic acid is the main fatty acid found in membrane lipids such as glycerophospholipids, where it is esterified as acyl residue to the glycerol part [45]. The addition of two acyl residues converts glycerol to diacylglycerol (DAG), which can be used for the synthesis of glycerophospholipids. DAG itself is a fusogenic lipid, because it induces a negative curvature in lipid bilayers [46,47]. It was shown that DAG can promote the fusion of protein-free liposomes in vitro [48,49], and therefore it has been suggested that these abilities might also be linked to efficient vacuole fusion in vivo [50]. Therefore, it can be assumed that the addition of oleic acid or glycerol in our experiments support the formation of DAGs, which then can bypass the *vac8*Δ caused block of vacuolar fusion.

In summary, we can show that oleic acid and glycerol each can complement the *vac8*Δ defect, and therefore can bypass the Vac8 requirement for vacuolar membrane fusion. Moreover, we describe that the biogenetic CPY pathway is independent of Vac8, while the degradative autophagic pathways of bulk autophagy, ribophagy, and pexophagy require Vac8 in *S. cerevisiae*.

Therefore, our data strongly suggest that the role of Vac8 in orchestrating vacuolar membrane dynamics is correlated with its function in autophagy.

## Supplementary Materials

The supplementary materials are available online https://www.mdpi.com/2073-4409/8/7/661/s1.
