# Supplementary material for: Vac8 Controls Vacuolar Membrane Dynamics during Different Autophagy Pathways in Saccharomyces cerevisiae
Source: Cells. 2019 Jun 30;8(7):661. doi: 10.3390/cells8070661 (PMC6678646; doi:10.3390/cells8070661)
Supplement: Supplementary file 1 [file cells-08-00661-s001.pdf]

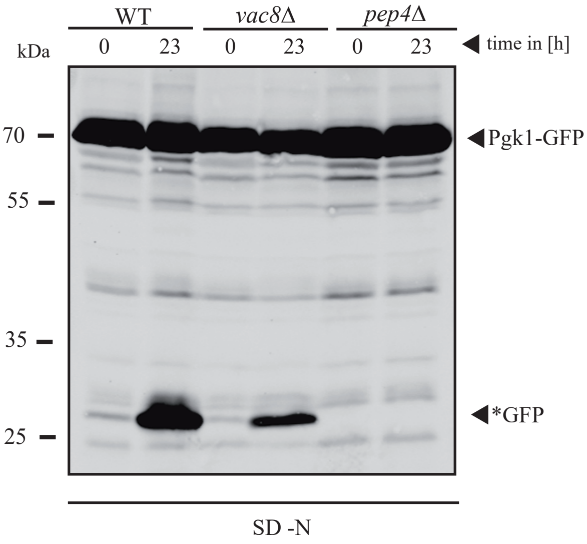

Figure S1. The degradation of the cytosolic protein Pgk1-GFP is slightly reduced under starvation conditions (SD-N) in *vac8Δ* cells.

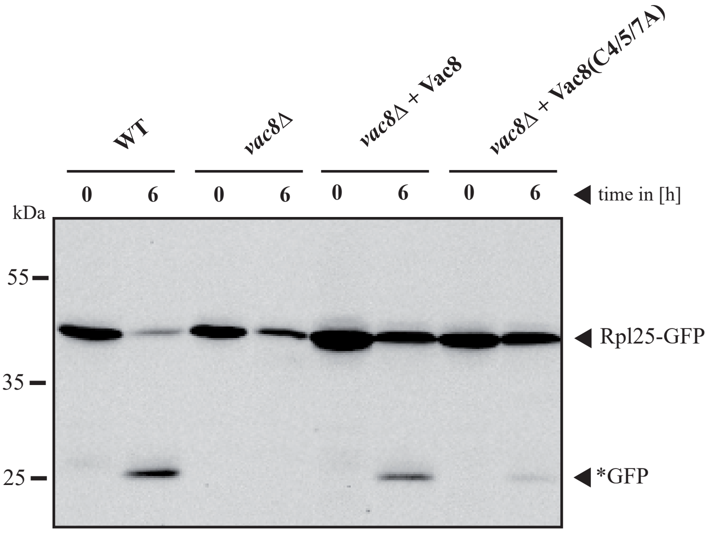

Figure S2. Palmitoylation of Vac8 is required for ribophagy. Ribosomes were marked with the Rpl25-GFP fusion protein. After induction of ribophagy, the appearence of free *GFP was detectable in WT and complemented cells, while it was hardly detectable in cells with the palmitoylation-deficient Vac8(C4/5/7A).

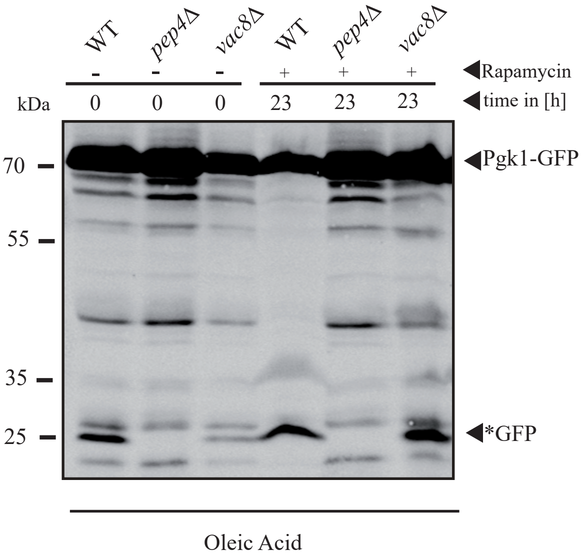

Figure S3. The impaired degradation of the cytosolic protein Pgk1-GFP in glucose-grown *vac8Δ* cells can be complemented by a shift to oleic acid-containing medium. This is shown by the WT-like level of free *GFP in *vac8Δ* cells after 23h.
